# A Biological Evaluation of Six Gene Set Analysis Methods for Identification of Differentially Expressed Pathways in Microarray Data

**DOI:** 10.4137/cin.s867

**Published:** 2008-06-20

**Authors:** Irina Dinu, Qi Liu, John D. Potter, Adeniyi J. Adewale, Gian S. Jhangri, Thomas Mueller, Gunilla Einecke, Konrad Famulsky, Philip Halloran, Yutaka Yasui

**Affiliations:** 1 School of Public Health, University of Alberta, 13-106 Clinical Sciences Building, Edmonton, AB, Canada T6G 2G3; 2 Division of Public Health Sciences, Fred Hutchinson Cancer Research Center, 1100 Fairview Ave. N., Seattle, WA, U.S.A. 98109; 3 Division of Nephrology and Transplantation Immunology, University of Alberta, 250 Heritage Medical Research Center, Edmonton, AB Canada T6G 2S2

## Abstract

*Gene-set analysis* of microarray data evaluates biological pathways, or gene sets, for their differential expression by a phenotype of interest. In contrast to the analysis of individual genes, gene-set analysis utilizes existing biological knowledge of genes and their pathways in assessing differential expression. This paper evaluates the biological performance of five gene-set analysis methods testing “self-contained null hypotheses” via subject sampling, along with the most popular gene-set analysis method, Gene Set Enrichment Analysis (GSEA). We use three real microarray analyses in which differentially expressed gene sets are predictable biologically from the phenotype. Two types of gene sets are considered for this empirical evaluation: one type contains “truly positive” sets that should be identified as differentially expressed; and the other type contains “truly negative” sets that should not be identified as differentially expressed. Our evaluation suggests advantages of SAM-GS, Global, and ANCOVA Global methods over GSEA and the other two methods.

## Introduction

Analytic methods of microarray data were initially formulated to identify *individual genes* that are differentially expressed according to a phenotype of interest[1]. Biological inference with microarray data, however, often focuses on the identification and interpretation of *pathways* (or *gene sets*) that are differentially expressed according to a phenotype. Prior to the publication of Gene Set Enrichment Analysis (GSEA) in 2003[2], such pathway-level inference was conducted unsystematically, often subjectively and manually by investigators going through the results of an individual-gene analysis. We would like to emphasize that the methods considered in this paper focus on identifying a-priori defined pathways, and not searching for statistically significant gene sets in Gene Ontology, by taking into account its hierarchical structure. However, gene set analysis methods considered here can be applied to a collection of a-priori defined gene sets from Gene Ontology.

GSEA proposed a systematic approach for evaluating gene sets for their differential expression between two classes of a phenotype. Using the Kolmogorov-Smirnov statistic, GSEA assesses the degree of “enrichment” of a set of genes (e.g. a pathway) in the entire range of the strength of associations with the phenotype of interest. GSEA has been modified in 2005[3] and has been used widely in gene-set analyses of microarray data. Following the proposal of GSEA, a number of gene-set analysis methods have been proposed.

The goal of this paper is to compare the performance of various gene-set analysis methods *biologically*. Our strategy for the biological comparison is to use microarray data with phenotypes that are known to be associated with certain gene sets (pathways). We used the 60 human cancer cell lines microarray expressions dataset (the NCI-60), assembled by the National Cancer Institute for anticancer drug discovery. To define the phenotype, we utilized the list of mutation status for 56 of the 60 cell lines provided for each of 24 genes studied by Ikediobi et al. [4]. We restricted our attention to genes where the mutation occurred in more than 10 cell-lines so that the performances of gene-set analysis methods can be compared in a statistically meaningful manner. There were four such genes among the 24 genes: 40 cell lines with mutant *p53* gene and 16 cell lines with wild-type *p53* gene; 31 cell lines with mutant Cyclin-dependent kinase inhibitor 2A (*CDKN2A* or *p16*) gene and 25 cell lines with wild-type *CDKN2A* gene; 11 cell lines with mutant Phosphatase and tension homologue (*PTEN*) gene and 45 cell lines with wild-type *PTEN* gene; 11 cell lines with mutant Kirsten rat sarcoma viral oncogene homolog (*KRAS*) gene and 45 cell lines with wild-type *KRAS* gene. For the *CDKN2A* mutation based phenotype, for example, pathways (or gene sets) that involve, or are closely related to, *CDKN2A* have a clear biological basis for being differentially expressed between the mutant-class and the wild-type-class of the phenotype of interest. On the other hand, pathways (or gene sets) that are far from *CDKN2A*’s functions have a biological basis for not being differentially expressed between the two classes of the phenotype. Using these pathways that are “biologically expected” or “biologically unexpected” to be associated with the phenotype, we can compare *sensitivity* (true positive rate) for identifying the “biologically expected” pathways as differentially expressed and *specificity* (true negative rate) for identifying the “biologically unexpected” pathways as not differentially expressed, across various gene-set analysis methods under the framework of Receiver Operating Characteristic (ROC) Analysis[5]. Here we used three microarray datasets corresponding to phenotypes defined by *CDKN2A*, *PTEN*, and *p53*. We did not study *KRAS*-defined phenotype comparison because there were only four biologically expected gene sets for *KRAS*, using the definition of “truly positive” gene sets described in Materials and Methods, which was insufficient for any statistical evaluation.

The recent review of gene-set analysis methods by Goeman and Bühlmann[6] provided an excellent summary of the methods. They made an important distinction among the gene-set analysis methods: those testing “self-contained null hypotheses” via. subject sampling and those testing “competitive null hypotheses” via. gene sampling. They pointed out that the competitive hypothesis testing via. gene sampling is subject to serious errors in calculating and interpreting statistical significance of gene sets, because of its implicit or explicit untenable assumption of stochastic independence across genes. Following the argument of Goeman and Bühlmann, we consider in this paper five methods that test “self-contained null hypotheses” via. subject sampling. We also include GSEA in the comparison as it is the most commonly-used method of gene-set analysis to date. The five methods that test “self-contained null hypotheses” via. subject sampling are: SAM-GS[7]; Global Test[8]; ANCOVA Global Test [9]; the method of Tian et al. [10]; the method of Tomfohr et al. [11]. Briefly, SAM-GS by Dinu et al. [7] is a gene-set analysis method that extended the individual-gene analysis method of SAM to gene-set analyses. It can also be seen as a modification of Dempster’s Test[13] for the two-sample multivariate mean comparison under a small-sample setting where the standard Hotelling’s T cannot be applied. Global Test was proposed by Goeman et al. [8] modeling and testing differential gene expression by use of random-effects logistic regression models. Mansmann and Meister[9] proposed ANCOVA Global Test in which the roles of phenotype and genes were exchanged in the regression modeling framework of Global Test. Tian et al. [10] assessed the significance of a gene set by taking the mean of t-test statistic values of genes in the gene set as a gene-set test statistic and evaluating its significance by a permutation of phenotype labels. Tomfohr et al. [11] reduced the gene set’s expression into a single summary value by taking the first principal component of expressions of genes in the gene set and performed a phenotype-label permutation test of the single summary. GSEA was initially proposed by Mootha et al. [2] using the Kolmogorov-Smirnov statistic to quantify the degree of “enrichment” of a set of genes in the entire range of the strength of associations with the phenotype. GSEA was later modified by Subramanian et al. [3]. Although GSEA is not a method for testing “self-contained null hypotheses” via. subject sampling, we included it here for comparison, as it is the most widely-used method of gene-set analyses.

For Global Test and ANCOVA Global Test, we previously found that it is necessary to standardize gene expression to eliminate the dominance within a gene-set by its member(s) with large variances [14]. We used the following equation to standardize the gene expression scores:

$$x_{jk}^{\prime }=\frac{x_{jk}-{\bar{x}}_{j}}{s_{j}},$$

where *x_jk_* is the gene expression for gene *j* in sample *k*, *x̄_j_* and *s_j_* are the mean value and standard deviation of gene *j* expression of all samples.

## Results

To compare the performance of the six methods, we used three gene-mutation based phenotypes (i.e. mutated vs. wild-type) in the NCI-60 microarray data: *CDKN2A; PTEN*; and *p53*. The gene sets accompanying the datasets were those used by Subramanian et al. [3]. For each of the three genes that defined the phenotype, we consider those gene sets containing that specific gene as “truly positive”, in the sense that a good gene set analysis method should identify those gene sets as being associated with the phenotype. For *CDKN2A*, there were 17 such gene sets. To define the “truly negative” gene sets, we search for genes whose frequency of appearance in the gene sets catalog was also 17, and identified 4 such genes. When searching PUBMED for links of each of these 4 genes with *CDKN2A*, we found that only *PRKACB* (protein kinase, cAMP-dependent, catalytic, beta) has no close link with *CDKN2A*, the gene defining the phenotype. We used the 17 gene sets, containing *PRKACB*, as “truly negative” gene sets. For the other two phenotypes, the “truly positive” and “truly negative” pathways were defined in the same way. For *PTEN*, there were 18 “truly positive” pathways; 5 other genes appeared 18 times in the gene sets catalog. We used *PRKAR2B* (protein kinase, cAMP-dependent, regulatory, type II, beta) to define the “truly negative” pathways, as we did not find any close links of this gene with *PTEN*, the gene defining the phenotype. For *p53*, there were 25 “truly positive” pathways; only one other gene appeared 25 times in the gene sets catalog: *RAC1* (ras-related *C3* botulinum toxin substrate 1). We used *RAC1* to define the “truly negative” pathways, as we did not find any close links of this gene with *p53*, the gene defining the phenotype. In the remaining of this section, we present the performance of the six gene set analysis methods on identifying these “truly positive” and “truly negative” gene sets.

### Identification of “truly positive” pathways: sensitivity of the methods

Table 1 shows the p-values of 17 “truly positive” pathways for differential expression by the phenotype according to the six gene-set analysis methods for *CDKN2A* mutation vs. wild-type comparison. SAM-GS, Global Test, and ANCOVA Global Test agreed closely and the majority of the 17 pathways showed statistically significant differential expression by the phenotype with p-value <0.05. On the other hand, the other three gene-set analysis methods identified only a few “truly positive” pathways with p-value <0.05. Similar results are found for *PTEN* mutation vs. wild-type, presented in Table 2, although SAM-GS found more significant gene sets than Global Test, and ANCOVA Global Test. The results for *p53* comparison, presented in Supplementary File, Table S1 online, agreed closely for SAM-GS, Global Test, and ANCOVA Global Test, and the majority of the 25 pathways showed statistically significant differential expression by the phenotype with p-value <0.05. On the other hand, the other three gene-set analysis methods identified only a few “truly positive” pathways with p-value <0.05.

### Identification of “truly negative” pathways: specificity of the methods

Table 3 shows the p-values of 17 “truly negative” pathways for differential expression by the phenotype, for *CDKN2A* mutation vs. wild-type comparison, according to the six gene-set analysis methods. Under the binomial assumption in a random sampling of 17 observations with p = 0.05 of “failure”, the probability observing 0, 1, 2, 3 “failure” in 17 observations are 0.42, 0.37, 0.16, and 0.04, respectively. None of the six gene-set analysis methods showed inconsistent results with the expected false positive numbers based on the binomial assumption. The results of the *PTEN* mutation vs. wild type analysis are presented in Table 4 for the 18 “truly negative” pathways for differential expression by the phenotype, according to the six gene-set analysis methods. Under the binomial assumption in a random sampling of 18 observations with p = 0.05 of “failure”, the probability observing 0, 1, 2, 3 “failure” in 18 observations are 0.40, 0.38, 0.17, and 0.05, respectively. None of the six gene-set analysis methods showed inconsistent results with the expected false positive numbers based on the binomial assumption. The results of the *p53* mutation vs. wild-type analysis are tabulated in Supplementary File, Table S2 online. Under the binomial assumption in a random sampling of 25 observations with p = 0.05 of “failure”, the probability observing 0, 1, 2, 3 “failure” in 25 observations are 0.28, 0.37, 0.23, and 0.09, respectively. None of the six gene-set analysis methods showed inconsistent results with the expected false positive numbers based on the binomial assumption.

### ROC analysis of the performance of the six gene-set analysis methods

Since the p-value threshold of 0.05 is arbitrary, we applied the ROC analysis to the classification of the “truly positive” and “truly negative” pathways that is independent of a specific choice of the threshold. That is, for each of the six gene-set analysis methods, we used the p-value for differential expression as the classifier of the two classes of pathways and evaluated the classification performance by the area under the ROC curve. Table 5 presents the areas under the ROC curves of the six methods and their associated 95% confidence intervals. The methods of Tian et al., Tomfohr et al., and GSEA had significantly smaller areas under the ROC curves compared to any of SAM-GS, Global Test, and ANCOVA Global Test (p < 0.05), indicating their significantly poorer performance in correctly classifying “truly positive” and “truly negative” pathways. The area under the ROC curve was above 0.80 for SAM-GS, Global Test, and ANCOVA Global Test, with each of them being significantly better than the random guess (p < 0.05). On the other hand, the area under the ROC curve was less than 0.65 for the other three methods, with each of them not being significantly different from the random guess. For the *PTEN* mutation vs. wild-type example, SAM-GS had significantly larger area under the ROC curves compared to Global (p < 0.05) and ANCOVA Global Tests (p < 0.05).

A smaller area under the ROC curve is expected for the un-standardized versions of the global tests. According to Qi et al. [14] the standardized versions of global tests are more powerful than the un-standardized versions, although the differences in performance may not be too large.

Any method that provides correct p-values can be used in conjunction with FDR methods such as the q-value method[15]. We included the FDR values for GSEA in our presentation, because it has a specific way of computing these values, and also because GSEA is the most popular method. Although we choose the p-values to rank the methods, we expect similar ranking based on the FDR values.

## Discussion

Proper evaluation of bioinformatics methods for microarray data analysis is not simple to perform. Simulation studies are useful for evaluating properties of methods under certain simplified conditions. It is, however, not possible to simulate complex correlations and noise properties that exist in real microarray data in measuring gene expression. On the other hand, evaluating data analysis methods empirically based on real microarray datasets is also subject to limitations, among which the most critical ones would perhaps be the question of generalizability of findings due to the use of specific datasets and not knowing what the underlying true expression profiles are. In this paper, we chose to evaluate the six gene-set analysis methods empirically using three analyses with the NCI-60 microarray data, each of the three corresponding to a phenotype defined based on mutated vs. wild-type of known cancer genes: *CDKN2A*, *PTEN*, and *p53.* Our goal was to compare gene-set analysis methods based on biological criteria. Although our strategy does not overcome the generalizability limitation, we were able to address the issue of unknown underlying expression profiles by utilizing the phenotype defined by mutation of a gene and analyzing biologically expected, and unexpected, differentially expressed gene sets.

The comparison of the six methods with respect to the true-positive and true-negative rates showed varying biological performance of these gene-set analysis methods, suggesting advantages of SAM-GS, Global, and ANCOVA Global methods over GSEA and the other two methods. These results are consistent to methodological features of the six methods. We provide two methodological remarks here that are relevant to the interpretation of the observed results. First, the six methods do not consider directions of the gene expression and the phenotype of interest in the same way. Both the method of Tian et al. and GSEA separate positive (over-expressed) and negative (under-expressed) associations of gene expression with the phenotype. That is, if some genes in a pathway are over-expressed and others are under-expressed by the phenotype, they work towards canceling each other in measuring the strength of association in these two methods. On the other hand, SAM-GS, Global Test, and ANCOVA Global Test take both directions of association as an indication of the association. Thus, pathways with a mixture of over-expressed and under-expressed genes are more likely to be identified as being significantly associated with the phenotype by these three methods than the method of Tian et al. or GSEA. The method of Tomfohr et al. initially reduces the gene expression of a pathway by taking the first principal component of the pathway’s gene expression *without considering the phenotype*. Thus, unless the direction of the first principal component is the direction along which the phenotype-associated expression differences appear, the method does not capture the phenotype-associated differential gene expression. Second, the six methods are not testing the same statistical null hypothesis. In the case of GSEA, for example, the null hypothesis tested is that genes of a pathway are not clustered along the axis of an association measure, such as correlation between the phenotype and gene expression[7]. This null hypothesis does not correspond to hypotheses of biological interest: if the genes of a pathway are clustered around the correlation values of zero, for instance, GSEA would still identify such pathways as being associated with the phenotype. In SAM-GS, Global Test, and ANCOVA Global Test, the null hypothesis is properly formulated statistically in three different ways.

There is a caveat in our approach to defining the “truly positive” and “truly negative” gene sets. In the *p53* analysis, for example, we took the gene sets that include *TP53* as “biologically expected truly positive” gene sets and those that include *RAC1* as “biologically expected truly negative” gene sets. We recognize that the appropriateness of the selection of these pathways may be debatable. In this example, there is one gene set, “arf pathway”, overlapping between the truly positive and truly negative gene sets. None of the six methods found evidence of significance for this pathway. There is no overlap in the *CDKN2A* analysis. In the *PTEN* analysis, “ins pathway” was listed as both “truly positive” and “truly negative”. *PTEN* is a tumour suppressor involved in cell cycle progression as an inhibitor of insulin (INS) signaling. In roundworms, a *PTEN* homologue has been related to development and longevity regulation through the INS-like pathway[12]. In addition to the overlapping issue, our definition of “truly positive” needs a remark. We considered gene sets as “truly positive” if they included the gene whose mutation defined the phenotype. For example, by including the *CDKN2A* gene that plays key biological functions in cell cycle regulations, the gene sets with *CDKN2A* are likely to be influenced by its mutation, at least partially, and, therefore, serve as biologically tenable “truly positive” gene sets. Note, however, that these gene sets may not necessarily be regulated primarily by the phenotype-defining gene.

In summary, our biological evaluation illustrated some appreciable performance differences among the six gene-set analysis methods. Our evaluation results are consistent throughout the three datasets in the sense that, SAM-GS, Global Test, and ANCOVA Global Test performed considerably better than the other three methods. More biologically-oriented evaluations of microarray analysis methods are needed, including those for gene-set analysis, for identifying truly effective gene-expression-analysis tools for biology and medicine.

## Materials and Methods

### The phenotypes being compared in the microarray dataset

To compare performance of the six methods, we obtained the NCI-60 microarray dataset from http://discover.nci.nih.gov/cellminer[16]. The microarray experiment of the NCI-60 was conducted by hybridizing 60 cancer cell lines’ mRNAs to Affy U95(A–E) chip. The expression data were normalized using RMA[17]. These arrays contain 49,064 ProbeSets and their expressions were reduced from the probe level to the gene level of 17,693 unique genes by a method described in the GSEA website [http://www.broad.mit.edu/gsea], by taking the maximum probe set expression of each gene in each sample. The mutation status of each cell line was based on the analysis of Ikediobi et al. [4]. According to Ikediobi et al. 59 of the 60 cell lines were made available, and a total of 56 independent cell lines were used for the mutation analysis: there were three pairs among the 59 cell lines that seemed to have been derived from the same individual. The synonymous pairs are the following: (a) “breast” cancer line NCI/ADR-RES and the ovarian cancer OVCAR-8, which have identical TP53 and ERBB2 variants and 99% genotype similarity; (b) the melanoma line M14 and the “breast” cancer line MDA-MB-435, which have identical BRAF, CDKN2A, and TP53 variants and 97% genotype similarity; and (c) two glioma lines SNB-19 and U251, which have identical TP53, CDKN2A, and PTEN variants and 96% genotype similarity. Lists of mutation status for each of the 56 cell lines were provided for each of 24 cancer genes studied by Ikediobi et al. [4]. We restricted our attention to four of the 24 genes where the mutation occurred in more than 10 cell-lines: *p53* (40 mutated vs. 16 wild-type); *CDKN2A* (31 mutate vs. 25 wild-type); *PTEN* and *KRAS* (both 11 mutated vs. 45 wild-type). Of these four genes, we did not study *KRAS*-defined phenotype comparison because there were only four “truly positive” gene sets for *KRAS*, using the definition of “truly positive” gene sets described in the next two subsections, which was insufficient for any statistical evaluation.

### Pathways/Gene sets

For gene sets, we used Subramanian et al.’s gene-set subcatalogs C2 from [http://www.broad.mit.edu/gsea] on “Molecular Signature Database.” In Subramanian et al. Catalog C2 consisted of 472 sets containing gene sets reported in manually curated databases and 50 sets containing genes reported in various experimental papers. Following the GSEA paper[3], we restricted the set size to be between 15 and 500, resulting in 310 pathways to be examined.

### Biological evaluation of gene-set methods

#### *CDKN2A* mutation vs wild-type

The phenotype of interest in this microarray experiment was defined by mutation of a specific gene, *CDKN2A*. Thus, gene sets that are expected on the basis of biology to be differentially expressed include those which involve *CDKN2A* gene as a gene set member. We considered gene sets that contain *CDKN2A* as a gene set member as “truly positive” gene sets. In the *CDKN2A* analysis, there were 17 such gene sets. A good gene-set analysis method should identify these gene sets as having differential expression between the mutant and wild-type classes. Regarding “truly negative” pathways, we searched the list of all genes on the microarray and identified 4 more genes whose frequency of appearances in the 310 pathways was also 17. A search on PUBMED indicated *PRKACB* (protein kinase, cAMP-dependent, catalytic, beta) as the only gene among the four with no close link with *CDKN2A*. We, therefore, used the 17 pathways that included *PRKACB* as “truly negative” pathways.

As a measure of gene-set analysis performance, statistical significance (p-value) of differential expression by the phenotype was computed for each of the 34 pathways by each of the six methods and tabulated. In the case of GSEA, which is not a method for testing “self-contained null hypotheses” via. subject sampling, both p-values and False Discovery Rate (FDR) provided by Subramanian et al. [3] were tabulated. In addition, we evaluated the classification performance of the six methods by the ROC analysis[4]. Conditioned on the fact that the 17 “truly positive” gene sets include *CDKN2A* as a member, we assumed conditional independence among the 17 gene sets and estimated the *sensitivity*, or true-positive rate, of classification (i.e. the proportion of the 17 “truly positive” gene sets which were correctly classified as truly positive), given a threshold of p-value. Similarly, conditioned on the fact that the 17 “truly negative” gene sets include *PRKACB* as a member, we assumed conditional independence among the 17 “truly negative” gene sets and estimated the *specificity*, or true-negative rate, of classification (i.e. the proportion of the 17 “truly negative” gene sets which were correctly classified as truly negative), given a threshold of p-value. By varying the threshold, we can draw an ROC curve, a curve that goes through points of (sensitivity, 1-specificity) across the whole range of classification threshold. The area under the ROC curve of each of the six methods was calculated by Mann-Whitney Statistic, and pairwise differences of the area under the ROC curve among the six methods were tested by the method of DeLong et al. [18]. Detailed calculations of the confidence intervals and pairwise differences tests are illustrated on a working example in the above mentioned reference. We used STATA to run these analyses.

#### *PTEN* mutation vs wild-type

The phenotype of interest in this analysis was defined by mutation of *PTEN*. We considered gene sets that contain *PTEN* as a member as “truly positive” gene sets. In the *PTEN* dataset, there were 18 such gene sets. Regarding “truly negative” gene sets, we searched the list of all genes on the microarray and identified 5 more genes whose frequency of appearances in the 310 pathways was also 18. A search on PUBMED indicated *PRKAR2B* (protein kinase, cAMP-dependent, regulatory, type II, beta) as the only gene among the five with no close link with *PTEN*. We, therefore, used the 18 gene sets that included *PRKAR2B* as “truly negative” gene sets. The ROC analysis was conducted analogous to the *CDKN2A* analysis.

#### *TP53* mutation vs wild-type

The phenotype of interest in this analysis was defined by mutation of *TP53*. We considered gene sets that contain *TP53* as a member as “truly positive” gene sets. In the *p53* analysis, there were 25 such gene sets. Regarding “truly negative” gene sets, we searched the list of all genes on the microarray and identified one more gene whose frequency of appearances in the 310 pathways was also 25, the same frequency as *TP53*. This gene was *RAC1* (ras-related *C3* botulinum toxin substrate 1) for which we did not find close link with *p53*. We, therefore, used the 25 pathways that included *RAC1* as “truly negative” gene sets. The ROC analysis was conducted analogous to the *CDKN2A* analysis.

## Contributions

ID and YY developed the SAM-GS methodology and designed/conducted the methodological study. TM, GE, KF, GJ, and PH performed the mouse transplant microarray study and introduced the gene-set analysis problem to ID and YY, including the GSEA methodology. JP provided biological interpretations of the analysis results of three real-world datasets. QL and AA contributed significantly to data analysis, refinement of SAM-GS, and programming. The manuscript was written primarily by ID, YY, and JP, and critically reviewed and revised by all authors. All authors read and approved the final manuscript.

## Supplementary Material

**Table S1:** P-values of the 25 pathways that include *TP53* gene by the six methods (with p-value or FDR < 0.05 in bold).

						GSEA	
Gene set	SAM-GS	Global	ANCOVA global	Tian	Tomfohr	P	FDR
atmPathway	<**0.000**	**0.008**	**0.007**	0.204	1.000	0.534	0.928
Cell_Cycle	<**0.000**	**0.005**	<**0.000**	<**0.000**	0.359	0.120	0.620
DNA_DAMAGE_SIGNALLING	<**0.000**	<**0.000**	<**0.000**	0.928	0.207	0.339	1.000
g1Pathway	<**0.000**	<**0.000**	<**0.000**	0.428	**0.037**	0.360	0.948
g2Pathway	<**0.000**	<**0.001**	<**0.001**	**0.012**	0.145	0.476	0.882
p53hypoxiaPathway	<**0.000**	**0.002**	<**0.001**	**0.028**	1.000	0.323	1.000
p53Pathway	<**0.000**	<**0.000**	<**0.000**	**0.010**	0.321	0.065	1.000
radiation_sensitivity	<**0.000**	0.006	**0.006**	0.270	1.000	0.342	1.000
SA_G1_AND_S_PHASES	<**0.000**	<**0.000**	<**0.000**	0.346	0.035	0.136	1.000
drug_resistance_and_metabolism	<**0.001**	**0.014**	**0.017**	0.020	1.000	0.128	1.000
p53_signalling	<**0.001**	**0.017**	**0.015**	0.818	1.000	0.855	0.956
CR_CELL_CYCLE	**0.002**	**0.009**	**0.005**	<**0.000**	0.399	0.340	0.886
chemicalPathway	**0.005**	0.052	**0.039**	0.980	1.000	0.291	0.899
breast_cancer_estrogen_signalling	**0.032**	0.111	0.123	0.564	1.000	0.540	0.904
ST_Fas_Signaling_Pathway	**0.043**	0.103	0.090	0.114	1.000	0.485	0.964
atrbrcaPathway	**0.050**	**0.048**	0.049	0.152	0.619	0.160	0.678
mitochondr	0.065	0.104	0.095	**0.048**	1.000	0.758	0.976
CR_DEATH	0.068	0.221	0.229	0.140	1.000	0.376	0.935
cell_cycle_checkpoint	0.122	0.119	0.106	**0.012**	0.855	**0.030**	0.420
tumor_supressor	0.187	0.173	0.177	**0.038**	1.000	0.167	0.883
RAP_UP	0.248	0.308	0.277	0.120	1.000	0.178	0.932
arfPathway	0.300	0.469	0.431	0.384	1.000	0.098	0.673
ST_JNK_MAPK_Pathway	0.579	0.707	0.705	0.362	1.000	0.790	0.951
telPathway	0.674	0.719	0.713	0.790	1.000	0.563	0.919
tidPathway	0.767	0.803	0.781	0.582	1.000	0.597	0.935

**Table S2:** P-values of the 25 pathways that include *RAC1* gene by the six methods (with p-value or FDR < 0.05 in bold).

						GSEA	
Gene set	SAM-GS	Global	ANCOVA global	Tian	Tomfohr	P	FDR
Raccycd Pathway	<**0.001**	**0.003**	**0.002**	0.560	0.997	0.192	0.968
actinY Pathway	**0.03+2**	0.074	0.067	0.092	0.970	0.119	1.000
NFKB_INDUCED	**0.033**	0.082	0.082	0.054	1.000	0.327	1.000
Ptdins Pathway	0.058	0.058	0.069	0.004	1.000	0.006	0.405
Creb Pathway	0.066	0.058	0.069	0.922	0.990	0.290	0.873
edg1 Pathway	0.134	0.155	0.148	0.558	1.000	0.658	0.934
Fml ppathway	0.135	0.200	0.193	0.006	1.000	0.023	0.578
Bcr Pathway	0.278	0.359	0.356	0.032	1.000	0.013	0.309
Arf Pathway	0.300	0.469	0.431	0.384	1.000	0.098	0.673
Cell_motility	0.369	0.430	0.455	0.456	1.000	0.205	0.891
G13_Signaling_Pathway	0.386	0.425	0.449	0.298	1.000	0.246	1.000
Wnt_Signaling	0.511	0.539	0.554	0.548	1.000	0.461	0.850
Mapk Pathway	0.609	0.683	0.649	0.750	1.000	0.940	0.981
SA_B_CELL_RECEPTOR_COMPLEXES	0.615	0.604	0.595	0.382	1.000	0.761	0.958
cell_adhesion	0.679	0.668	0.675	0.264	1.000	0.964	0.970
Tcr Pathway	0.709	0.779	0.772	0.036	1.000	0.453	0.853
p38mapk Pathway	0.715	0.762	0.765	0.376	1.000	0.222	0.996
Ucalpain Pathway	0.750	0.760	0.758	0.488	1.000	0.825	0.983
pyk2 Pathway	0.779	0.863	0.846	0.230	1.000	0.013	0.477
rac1 Pathway	0.785	0.720	0.707	0.264	1.000	0.697	0.965
CR_CAM	0.827	0.880	0.884	0.448	1.000	0.917	0.968
ST_MONOCYTE_AD_PATHWAY	0.884	0.889	0.901	0.878	1.000	0.359	0.851
Ras Pathway	0.911	0.921	0.912	0.642	1.000	0.895	1.000
at1rPathway	0.954	0.971	0.974	0.624	1.000	0.165	0.887
nkcellsPathway	0.974	0.992	0.986	0.872	1.000	0.939	0.991

## Figures and Tables

**Table 1:** P-values of the 17 pathways that include *CDKN2A* gene by the six methods (with p-value or FDR < 0.05 in bold).

						GSEA	
Gene set	SAM-GS	Global	ANCOVA global	Tian	Tomfohr	P	FDR
arfPathway	<**0.001**	**0.004**	**0.005**	0.284	1.000	0.143	0.807
breast_cancer_estrogen_signalling	<**0.001**	**0.002**	**0.009**	0.506	0.750	0.443	0.824
cell_cycle_arrest	<**0.001**	0.053	0.056	0.174	1.000	0.904	0.892
cellcyclePathway	<**0.001**	**0.001**	<**0.001**	0.926	1.000	0.371	0.638
g1Pathway	<**0.001**	<**0.001**	<**0.001**	0.476	1.000	0.190	0.549
SA_G1_AND_S_PHASES	<**0.001**	**0.012**	**0.011**	0.164	1.000	0.163	0.824
SIG_PIP3_signaling_in_B_lymphocytes	<**0.001**	**0.006**	**0.005**	0.898	1.000	0.497	0.815
SIG_PIP3SIGINCARDIACMYOCTES	<**0.001**	**0.011**	**0.009**	0.712	1.000	0.285	0.605
ST_Phosphoinositide_3_Kinase_Pathway	<**0.001**	**0.004**	**0.004**	0.822	1.000	0.122	0.743
SIG_InsulinReceptorPathwayIn CardiacMyocytes	**0.001**	**0.019**	**0.015**	0.218	0.999	0.249	0.608
p53_signalling	0.002	0.042	0.036	0.010	1.000	0.038	0.594
ST_Integrin_Signaling_Pathway	**0.005**	**0.045**	**0.036**	**0.028**	1.000	0.175	0.515
HTERT_UP	**0.011**	0.056	0.056	0.164	1.000	0.510	0.703
drug_resistance_and_metabolism	**0.013**	0.104	0.102	0.822	1.000	0.526	0.789
CR_CELL_CYCLE	**0.018**	0.116	0.109	0.600	1.000	0.623	0.757
Cell_Cycle	**0.019**	0.103	0.091	0.160	1.000	0.720	0.852
PROLIF_GENES	**0.049**	0.097	0.070	0.246	1.000	0.404	0.645

**Table 2:** P-values of the 18 pathways that include *PTEN* gene by the six methods (with p-value or FDR < 0.05 in bold).

						GSEA	
Gene set	SAM-GS	Global	ANCOVA global	Tian	Tomfohr	P	FDR
igf1mtorPathway	<**0.001**	**0.032**	**0.019**	0.258	0.808	0.141	0.987
ptenPathway	<0.001	0.008	0.006	0.012	1.000	0.000	0.106
SA_PTEN_PATHWAY	<0.001	0.003	0.008	0.014	1.000	0.023	0.734
tumor_supressor	<**0.001**	**0.014**	**0.016**	0.876	1.000	0.482	0.956
INS	0.002	0.031	0.022	0.004	1.000	0.038	0.664
eif4Pathway	**0.003**	**0.038**	**0.037**	0.280	0.987	0.717	0.979
mtorPathway	**0.003**	0.113	0.123	0.302	1.000	0.204	0.974
SIG_PIP3_signaling_in_B_lymphocytes	**0.005**	0.061	0.058	0.222	1.000	0.548	0.936
metPathway	**0.008**	**0.034**	**0.039**	0.806	1.000	0.732	0.977
ST_Phosphoinositide_3_Kinase_Pathway	**0.008**	0.080	0.066	0.276	1.000	0.116	1.000
SIG_CHEMOTAXIS	**0.014**	0.085	0.073	0.588	1.000	0.721	0.964
SIG_InsulinReceptorPathwayIn CardiacMyocytes	**0.015**	0.076	0.072	0.120	1.000	**0.034**	1.000
ST_Integrin_Signaling_Pathway	**0.023**	0.076	0.066	0.538	1.000	0.915	0.976
SIG_PIP3SIGINCARDIACMYOCTES	0.092	0.318	0.320	0.130	1.000	0.216	1.000
cell_proliferation	0.127	0.170	0.171	0.950	1.000	0.409	0.894
PROLIF_GENES	0.143	0.222	0.225	0.310	1.000	0.377	0.914
CR_SIGNALLING	0.154	0.299	0.302	0.042	1.000	0.278	1.000
breast_cancer_estrogen_signalling	0.207	0.438	0.423	0.472	1.000	0.780	1.000

**Table 3:** P-values of the 17 pathways that include *PRKACB* gene by the six methods (with p-value or FDR < 0.05 in bold).

						GSEA	
Gene set	SAM-GS	Global	ANCOVA global	Tian	Tomfohr	P	FDR
crebPathway	0.022	0.026	0.032	0.006	1.000	0.009	0.409
gpcrPathway	**0.036**	0.057	0.058	0.122	1.000	**0.041**	0.495
pparaPathway	0.070	0.087	0.083	**0.016**	1.000	0.079	0.471
nos1Pathway	0.119	0.138	0.132	0.350	1.000	0.428	0.649
badPathway	0.124	0.148	0.134	0.144	1.000	0.082	0.617
gata3Pathway	0.130	0.224	0.204	0.446	1.000	0.292	0.605
amiPathway	0.131	0.133	0.119	**0.014**	0.998	0.285	0.533
cskPathway	0.131	0.133	0.119	**0.014**	0.998	0.285	0.525
chrebpPathway	0.135	0.209	0.193	0.286	1.000	0.353	0.597
no1Pathway	0.166	0.260	0.283	**0.050**	1.000	0.078	0.557
ck1Pathway	0.243	0.372	0.373	0.428	1.000	0.291	0.564
mprPathway	0.243	0.285	0.263	0.886	0.999	0.591	0.760
mcalpainPathway	0.283	0.383	0.369	0.060	1.000	0.146	0.509
shh_lisa	0.317	0.361	0.362	0.880	1.000	0.431	0.656
CR_PROTEIN_MOD	0.368	0.405	0.392	0.362	1.000	0.395	0.647
nfatPathway	0.424	0.534	0.556	0.352	1.000	0.198	0.529
vipPathway	0.449	0.546	0.569	**0.026**	1.000	0.309	0.601

**Table 4:** P-values of the 18 pathways that include *PRKAR2B* gene by the six methods (with p-value or FDR < 0.05 in bold).

						GSEA	
Gene set	SAM-GS	Global	ANCOVA global	Tian	Tomfohr	P	FDR
INS	0.002	0.031	0.022	0.004	1.000	0.038	0.664
amiPathway	**0.042**	0.054	**0.032**	**0.002**	1.000	0.117	0.777
cskPathway	**0.042**	0.054	**0.032**	**0.002**	1.000	0.117	0.743
mprPathway	0.128	0.180	0.161	0.148	0.984	0.138	0.756
gpcrPathway	0.217	0.202	0.169	0.386	1.000	0.063	0.661
no1Pathway	0.355	0.270	0.248	0.528	1.000	0.642	0.931
nfatPathway	0.386	0.335	0.347	0.396	1.000	0.898	0.948
vipPathway	0.403	0.350	0.328	0.462	1.000	0.394	0.970
nos1Pathway	0.413	0.454	0.431	0.926	1.000	0.153	0.744
mcalpainPathway	0.621	0.629	0.601	0.190	1.000	0.625	1.000
pparaPathway	0.665	0.674	0.668	0.130	1.000	0.549	0.934
shh_lisa	0.671	0.689	0.661	0.294	1.000	0.496	0.963
ck1Pathway	0.704	0.758	0.762	0.174	1.000	0.123	0.793
crebPathway	0.732	0.798	0.806	0.708	1.000	0.119	0.756
chrebpPathway	0.745	0.809	0.804	0.232	1.000	0.155	0.753
CR_PROTEIN_MOD	0.760	0.805	0.788	0.852	1.000	0.608	0.920
badPathway	0.830	0.876	0.854	0.480	1.000	0.747	0.948
gata3Pathway	0.921	0.883	0.880	0.522	1.000	0.555	0.933

**Table 5:** ROC analysis comparing the six methods for each of the three microarray datasets.

Phenotype	Gene-set analysis method	Area under the ROC curve (95% Confidence Interval)
*CDKN2A* mutation vs. wild type	SAM-GS	0.993 (0.977, 1.000)
	Global	0.945 (0.873, 1.000)
	ANCOVA Global	0.948 (0.879, 1.000)
	Tian*	0.360 (0.163, 0.557)
	Tomfohr*	0.545 (0.396, 0.694)
	GSEA p-value*	0.395 (0.195, 0.594)
	GSEA FDR*	0.180 (0.038, 0.321)
*PTEN* mutation vs. wild type	SAM-GS	0.918 (0.822, 1.000)
	Global	0.847 (0.715, 0.979)
	ANCOVA Global	0.832 (0.691, 0.972)
	Tian*	0.506 (0.309, 0.703)
	Tomfohr*	0.528 (0.435, 0.620)
	GSEA p-value*	0.500 (0.301, 0.699)
	GSEA FDR*	0.265 (0.086, 0.445)
*P53* mutation vs. wild type	SAM-GS	0.854 (0.752, 0.957)
	Global	0.828 (0.714, 0.942)
	ANCOVA Global	0.831 (0.718, 0.944)
	Tian*	0.625 (0.464, 0.785)
	Tomfohr*	0.647 (0.525, 0.769)
	GSEA p-value*	0.529 (0.361, 0.697)
	GSEA FDR*	0.482 (0.315, 0.649)

* These methods have significantly smaller area under the ROC curve compared to SAM-GS, Global, or ANCOVA Global methods (p < 0.05).

## Abbreviations

GSEA: Gene Set Enrichment Analysis
SAM-GS: Significance Analysis of Microarrays for Gene Sets
ROC: Receiver Operating Characteristic
